# The In Vitro Antibacterial Activity of Argirium SUNc against Most Common Pathogenic and Spoilage Food Bacteria

**DOI:** 10.3390/antibiotics13010109

**Published:** 2024-01-22

**Authors:** Andrea Mancusi, Marica Egidio, Raffaele Marrone, Luca Scotti, Domenico Paludi, Irene Dini, Yolande Thérèse Rose Proroga

**Affiliations:** 1 Department of Food Safety Coordination, Istituto Zooprofilattico Sperimentale del Mezzogiorno, 80055 Portici, Italy; andrea.mancusi@izsmportici.it (A.M.); yolande.proroga@izsmportici.it (Y.T.R.P.); 2 Department of Veterinary Medicine and Animal Production, University of Naples Federico II, 80138 Naples, Italy; marica.egidio@libero.it (M.E.); raffaele.marrone@unina.it (R.M.); 3 Department of Medical, Oral, and Biotechnological Sciences, “G. d’Annunzio” University of Chieti–Pescara, 66100 Chieti, Italy; 4 Faculty of Veterinary Medicine, University of Teramo, 64100 Teramo, Italy; dpaludi@unite.it; 5 Department of Pharmacy, University of Naples Federico II, Via Domenico Montesano 49, 80131 Naples, Italy; irdini@unina.it

**Keywords:** antimicrobial activity, silver ultra nanoclusters SUNc, multi-antibiotic-resistant bacteria

## Abstract

Foodborne diseases are one of the main issues for human health, and antibacterial packaging plays a major role in food security assurance. Silver ultra nanoparticles (Argirium SUNc) are antimicrobial agents that have a wide spectrum of action, including against pathogenic bacteria and spoilage fungi. The aim of the present study was to evaluate the antibacterial activity of Argirium SUNc on the bacteria most commonly found in food: *Staphylococcus aureus*, *Pseudomonas aeruginosa*, *Escherichia coli*, *Listeria monocytogenes*, and *Salmonella typhimurium*. In this regard, an in vitro study was carried out by assessing the Argirium SUNc effectiveness on different concentrations of each tested microbial strain and at different time intervals. The data showed that the antimicrobial activity of Argirium SUNc was directly related to the microbial concentration and varied depending on the microbial species. Moreover, a greater effectiveness against Gram-negative bacteria than Gram-positive bacteria was observed. These preliminary results provided important information on the silver nanoparticles spectrum of action, and this is an aspect that appears particularly promising for obtaining a viable alternative to traditional antimicrobials to be used against the pathogens and spoilage agents most commonly found in the food chain, harmful both to health and quality aspects.

## 1. Introduction

Microbial contamination, which can occur at any stage of the food production, delivery, and consumption chain, is one of the main problems of the food industry, considering the implications for public health due to foodborne diseases [1]. Foodborne illnesses (TADs) are linked to the ingestion of water or food contaminated by pathogenic microorganisms such as bacteria, viruses, or parasites, and occur only if their toxins/metabolites reach the minimal infectious dose [2]. Therefore, the ingestion of harmful, unhealthy, and unsafe food represents a risk for the consumer and, as such, can have significant consequences on their health. Data published by the World Health Organization (WHO) reveal that “1,600,000 people get sick due to unsafe food in one day, on average and 340 children under 5 years of age die due to preventable foodborne diseases, on average, every day” [3]. For this reason, food quality assurance systems applied to production processes to inactivate the growth of undesirable microorganisms are essential for public health protection. In this context, different types of antibiotic agents in food or in packaging have been used; however, bacteria have evolved by developing defense mechanisms that allow them to survive and proliferate even in the presence of an antibiotic. This phenomenon, called *antibiotic resistance*, is due to many complex mechanisms of action [4] resulting from spontaneous genetic mutations or acquired through the exchange of genetic material with individuals of the same species or of different species. An example can be represented by proteins or enzymes that lead to drug alteration, causing it to lose its biological function; upregulated efflux pumps, which remove drugs from the bacterial cell; biofilm production, which is an aggregate of microorganisms enclosed in a self-produced polymeric matrix [5], allowing them to survive an antibiotic attack; or even genes that modify drug targets [6,7], reducing or deactivating antibiotic effectiveness. Furthermore, many antibiotics have the same mechanism of action, which is why microorganisms can develop multiple resistances against the entire class of molecules [8]. For this reason, common bacterial infections that are now possible to treat, in future, could be responsible for numerous deaths. Because of this, nowadays, antibiotic resistance poses one of the biggest global threats for human and animal health, having an impact on the medical, veterinary, and agricultural sectors [9,10,11]. Consequently, finding alternative substances to available antibiotics with high antimicrobial activity and different mechanisms of action is a major global challenge for the life sciences community. In the food industry, but also in the medical field or the livestock sector, nanomaterials may offer potential solutions for this challenge. In particular, silver ultra nanoparticles (SUNc), innovative and biocompatible solutions, are possible candidates for antibacterial activity, having a wide spectrum of action, including against pathogenic bacteria and spoilage fungi, which allows them to significantly reduce contamination and improve the hygienic-sanitary quality of food products.

### Silver Nanoparticles

The term nanoparticle means an engineered structure with sizes ranging from 1 to 100 nm [12], whose properties are influenced by a number of factors, including chemical composition, shape, size, and size distribution [13]. They usually consist of noble metals and/or metal oxides such as Ag, Au, Cu, CuO, TiO_2_, and MgO, etc. [14] and can be produced using two different methods:

- *The top-down method*, which is a physical method that involves the breakdown of a bulk material into smaller parts through simple grinding. It is easily applicable, economical, and does not require the use of volatile and toxic compounds. However, the quality of the nanoparticles produced by this method is lower than that of nanocomposites made with the modern bottom-up method, as there might be contamination problems related to milling equipment, irregular shape and size, and high demands [15];
- *The bottom-up method*, which is a chemical method that involves the use of atomic or molecular raw materials that must be chemically converted into larger nanoparticles. It is expensive, but much more controllable and accurate than the top-down method and, for this reason. the nanoparticles produced using the bottom-up technique, chemically based and designed, are qualitatively better.

Regardless of how they are created, all nanoparticles must be stabilized, that is, deposited/embedded in a solid substrate or host material that must be suitable for the applications for which they were designed. The best solution for the stabilization of these particles is represented by their inclusion in polymers with the formation of metal–polymer nanocomposites [16]. There are two ways to obtain these products:

- The in situ *method*, where the monomer is polymerized and the metal ions, which are reduced in the polymer matrix to form nanoparticles, may be introduced before or after polymerization;
- The ex situ *method*, in which nanoparticles are first synthesized and then introduced into a polymeric solution or a monomer that is subsequently polymerized.

Among metal nanoparticles, AgNps are the most studied and utilized [17], mainly due to their antimicrobial properties that have been known since ancient times, when silver and its derivates were used for food preservation, water sanitization [18,19], or even for healing burn wounds [20]. Numerous studies have shown that AgNps have good biocidal activity against both Gram-negative and Gram-positive bacteria [21], some fungi, and some viruses [22]. On the contrary, unlike traditional antibiotics, they have a low propensity to induce microbial resistance [23], probably because they have the advantage of acting on multiple targets [24]. Their antibacterial activity is due to a combination of multiple mechanisms of action, some linked to the silver ions continuously released by the particle, others to the nanoparticle itself [25]:

- The nanoparticle is able to attack and break the cell membrane, altering its permeability. Following this penetration, it can also damage the internal cellular compartments, affecting vital functions because it acts on the respiratory chain, blocking bacterial respiration and ATP synthesis, so as to inhibit cell division and cause the death of the cell itself. Furthermore, AgNPs induce the production of reactive oxygen species (ROS), which lead to cellular oxidative stress and apoptosis [26];
- Instead, Ag^+^ ions have the ability to easily bind to the amino (-NH_2_), imidazole (CH_2_)_2_N(NH)CH, carbonyl (C=O), phosphate R-OPO(OH)_2_, and thiol groups (R-SH), altering common biological functionalities. They are also able to form complexes with peptides and nucleic acids (DNA or RNA), inhibiting their transcription [27] and interfering with the cell replication process [28].

Moreover, the antimicrobial properties of AgNPs are also linked to their shape and size. Data have shown that silver nanocomposites in the size range of 1–10 nm have the greatest antibacterial activity, because a smaller nanoparticle penetrates more easily in a bacterial cell membrane and dissolves faster, releasing more Ag^+^ [29]. Beyond size, shape also influences the antibacterial performance of AgNPs, which depends on how good the contact is between the nanomaterial and the bacterial cell membrane. For this reason, there are four morphology parameters that play important roles in the antibacterial effectiveness of AgNPs: the geometry of interaction, surface area, crystal facets, and sharpness of the edges [30].

The aim of the present work was to evaluate the antimicrobial activity of silver ultra nanoclusters (Argirium SUNc^®^) at different concentrations and at different time intervals, against the most isolated bacteria in foods: *Staphylococcus aureus*, *Pseudomonas aeruginosa*, *Escherichia coli*, *Listeria monocytogenes*, and *Salmonella enterica serovar typhimurium*.

## 2. Results

The present study, relating to the evaluation of Argirium SUNc^®^ antimicrobial activity, provided us with preliminary data regarding the presence and intensity of their inhibitory action against tested microorganisms. It was observed that the antimicrobial activity of nanocomposites varied depending on the microbial species and took on an intensity that appeared to be inversely proportional to their concentration level (except for *Listeria monocytogenes*, Table 1). Furthermore, the studied Gram-negative bacteria showed a slightly higher sensitivity to silver preparation compared to Gram-positive microorganisms. 

### 2.1. Determination of Argirium SUNc^®^ Antibacterial Effect against Listeria monocytogenes and Staphylococcus aureus

**Figure 1:**
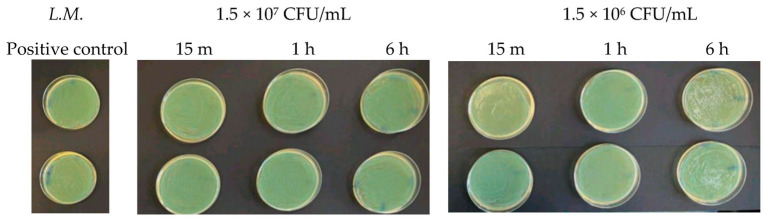
Antibacterial effect of Argirium SUNc^®^ against *Listeria monocytogenes* at concentrations of 1.5 × 10^7^ CFU/mL and 1.5 × 10^6^ CFU/mL.

**Figure 2:**
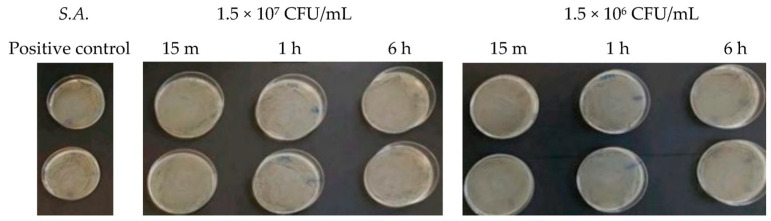
Antibacterial effect of Argirium SUNc^®^ against *Staphylococcus aureus* at concentrations of 1.5 × 10^7^ CFU/mL and 1.5 × 10^6^ CFU/mL.

At microbial concentrations equal to 1.5 × 10^7^ CFU/mL and 1.5 × 10^6^ CFU/mL, no inhibitory effect of Argirium SUNc^®^ was observed. In fact, the agar plates presented a patina that is a sign of considerable difficulty for the nanostructures to act and an index of the absence of a real inhibitory action for the different incubation times considered. Figure 1 (in which there is *Listeria monocytogenes*) and Figure 2 (in which there is *Staphylococcus aureus)* show that the bacterial cells treated with the nanoparticles reached CFU/mL levels comparable to those of the untreated bacteria (positive control). 

**Figure 3:**
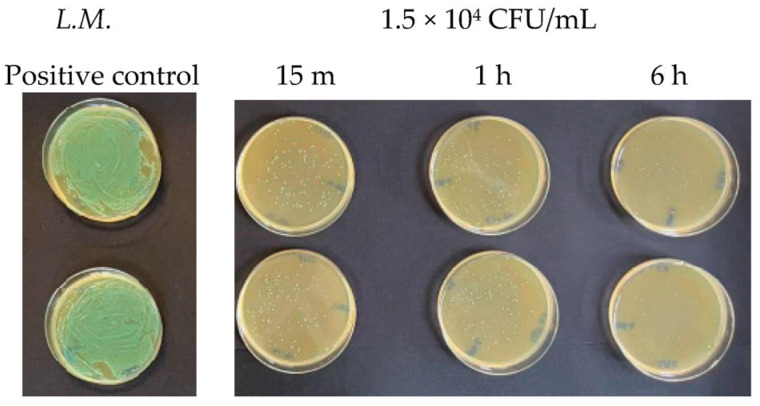
Antibacterial effect of Argirium SUNc^®^ against *Listeria monocytogenes* at a concentration of 1.5 × 10^4^ CFU/mL.

**Figure 4:**
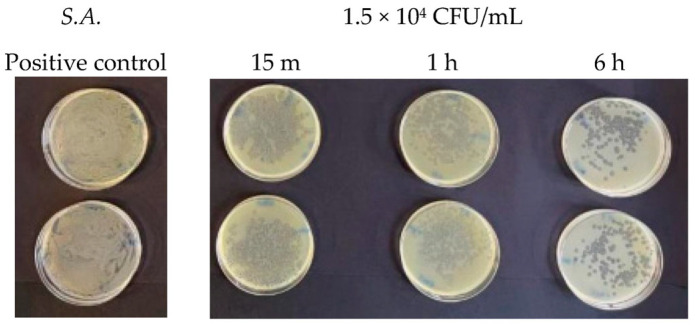
Antibacterial effect of Argirium SUNc^®^ against *Staphylococcus aureus* at a concentration of 1.5 × 10^4^ CFU/mL.

When the microbial concentration was 1.5 × 10^4^ CFU/mL, Argirium SUNc^®^ showed their effectiveness, inactivating both Gram-positive bacteria tested within 15 min (Figure 3 and Figure 4), with inhibition percentages (for each tested microorganism, the inhibitory power was calculated using the following equation: after colony counting, having two agar plates for each test, the average between the two numbers (m) was obtained. Based on the number of surviving germs, with a microbial concentration equal to 1.5 × 10^2^ CFU/mL, the antibacterial activity of the silver nanoparticles was determined using the following proportion: 1.5 × 10^2^:100= m:x; with a microbial concentration equal to 1.5 × 10^2^ CFU/mL, the antibacterial activity of the silver nanoparticles was determined using the following proportion: 1.5 × 10^4^:100 = m:x. Finally, the AgNPs’ inhibitory power (%) was calculated by subtracting the proportion result from 100 (inhibitory power % = 100-result proportion)) of 99.3% against *Listeria monocytogenes* (Table 1) and 96.9% for *Staphylococcus aureus* (Table 2). This antimicrobial activity had an increasing trend over time with an almost total inhibition of microbial proliferation (99.8% for *Listeria monocytogenes* and 98.59% for *Staphylococcus aureus*) after 6 h. 

**Figure 5:**
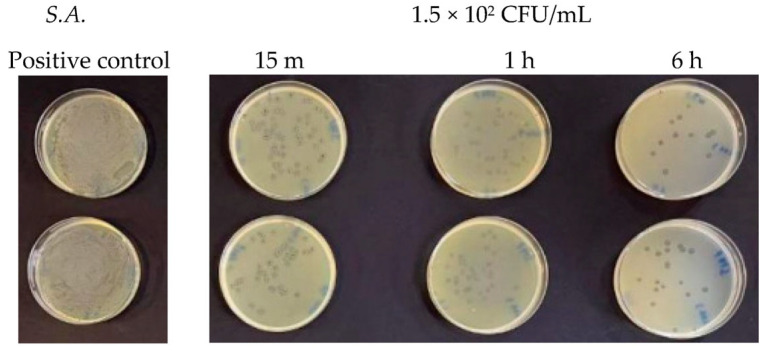
Antibacterial effect of Argirium SUNc^®^ against *Staphylococcus aureus* at a concentration of 1.5 × 10^2^ CFU/mL.

**Figure 6:**
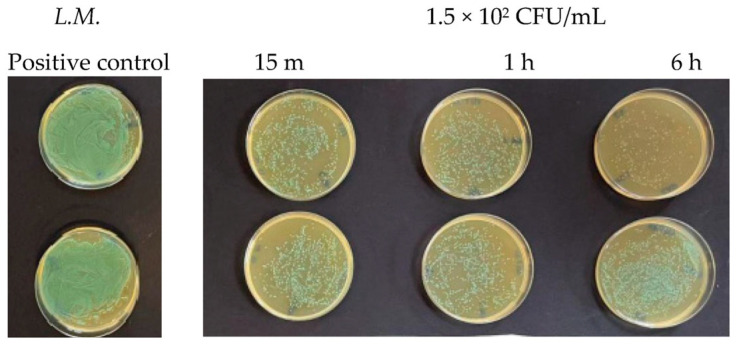
Antibacterial effect of Argirium SUNc^®^ against *Listeria monocytogenes* at a concentration of 1.5 × 10^2^ CFU/mL.

Finally, at a concentration of 1.5 × 10^2^ CFU/mL, two different behaviors were observed. For *Staphylococcus aureus*, a very high reduction in the number of viable bacteria was observed compared to the untreated control after 15 min (Figure 5). This bactericidal action continued over time and increased after 1 h and even more after 6 h, as shown in Table 2. *Listeria monocytogenes* showed a greater resistance towards Argirium SUNc^®^ at various incubation times (15 min, 1 h, and 6 h) compared to *Staphylococcus aureus*. This condition can be observed on the agar plates in Figure 6 and in Table 1, in which by calculating the inhibition percentage (%), it was seen that Argirium SUNc^®^ presented poor antibacterial activity with an inhibitory power that, in some cases, was less than 50% (Table 1).

### 2.2. Determination of Argirium SUNc^®^ Antibacterial Effect against Pseudomonas aeruginosa, Escherichia coli, Salmonella typhimurium Isolated from Mussels, and Salmonella typhimurium Isolated from Humans

Gram-negative bacteria had different antibacterial responses to the Argirium SUNc^®^ action and showed a higher sensitivity compared to Gram-positive microorganisms. 

**Figure 7:**
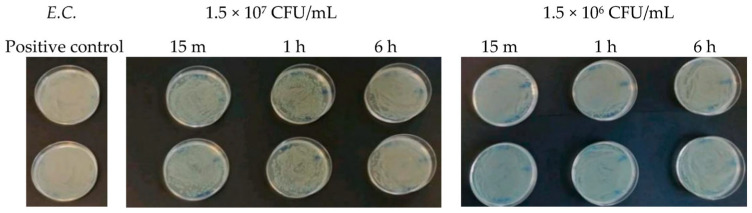
Antibacterial effect of Argirium SUNc^®^ against *Escherichia coli* at concentrations of 1.5 × 10^7^ CFU/mL and 1.5 × 10^6^ CFU/mL.

**Figure 8:**
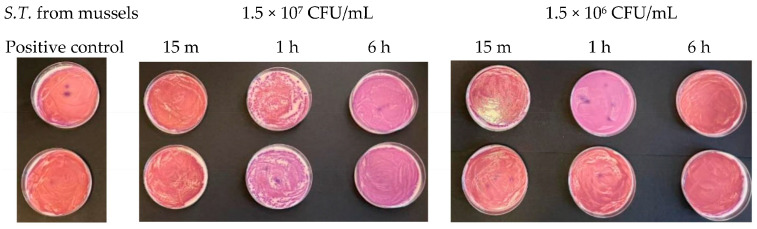
Antibacterial effect of Argirium SUNc^®^ against *Salmonella typhimurium* isolated from mussels at concentrations of 1.5 × 10^7^ CFU/mL and 1.5 × 10^6^ CFU/mL.

**Figure 9:**
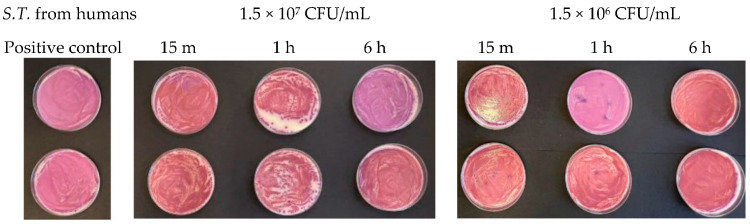
Antibacterial effect of Argirium SUNc^®^ against *Salmonella typhimurium* isolated from humans at concentrations of 1.5 × 10^7^ CFU/mL and 1.5 × 10^6^ CFU/mL.

**Figure 10:**
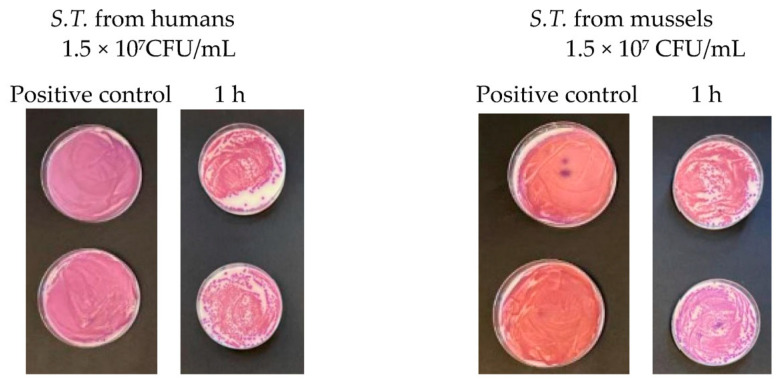
Comparison between *Salmonella typhimurium* isolated from humans and *Salmonella typhimurium* isolated from mussels.

**Figure 11:**
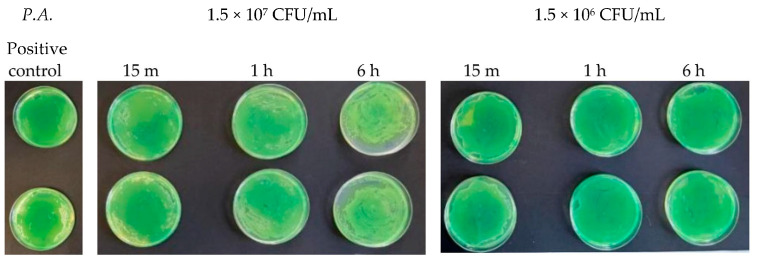
Antibacterial effect of Argirium SUNc^®^ against *Pseudomonas aeruginosa* at concentrations of 1.5 × 10^7^ CFU/mL and 1.5 × 10^6^ CFU/mL.

At a concentration equal to 1.5 × 10^7^ CFU/mL, in the agar plates containing *Escherichia coli*, inhibitory powers (estimated based on the reduction in the number of viable bacteria compared to the untreated positive control) of around 5% after 15 min and around 10% after 1 h were observed. However, after 6 h, the tested microorganism underwent progressive regrowth, taking over the action of the Argirium SUNc^®^; therefore, the estimated inhibitory power corresponded to less than 1% (Figure 7). For the *Salmonella typhimurium* strain isolated from mussels and the *Salmonella typhimurium* strain isolated from humans, Argirium SUNc^®^ antibacterial activity was observed only after 1 h (Figure 8 and Figure 9), as after 15 min and after 6 h, no inhibition occurred and the number of CFU/mL of these microorganisms was approximately the same number of bacteria as those of the untreated (positive control). Finally, in the agar plates containing *Pseudomonas aeruginosa* at a concentration of 1.5 × 10^7^ CFU/mL, a decrease in bacterial growth was observed only after 6 h (Figure 11). It is important to underline the difference in behavior observed between the *Salmonella typhimurium* strain isolated from mussels and that isolated from humans: this one, at a concentration of 1.5 × 10^7^ CFU/mL, presented a greater sensitivity against Argirium SUNc^®^ compared to that isolated from humans, as demonstrated in Figure 10 (the inhibition percentage of Argirium SUNc^®^ is greater). 

In the suspension with a density of 1.5 × 10^6^ CFU/mL, Argirium SUNc^®^ showed poor antibacterial activity against *Escherichia coli* and *Pseudomonas aeruginosa*, with a decrease in bacterial growth only after 6 h (Figure 7 and Figure 11). Slight inhibitory activity against *Salmonella typhimurium* isolated from mussels and *Salmonella typhimurium* isolated from humans after 15 min was observed. However, after 1 h for *Salmonella typhimurium* isolated from mussels (Figure 8) and after 6 h for *Salmonella typhimurium* isolated from humans (Figure 9), microorganisms resumed their growth, rendering Argirium SUNc^®^ ineffective.

**Figure 12:**
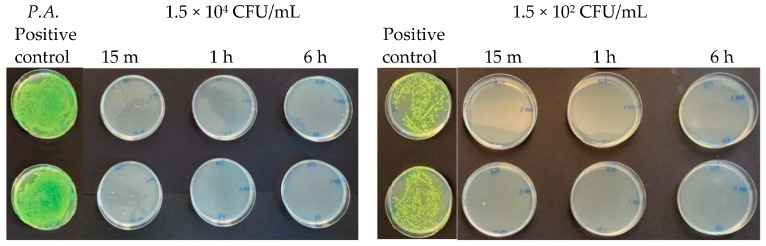
Antibacterial effect of Argirium SUNc^®^ against *Pseudomonas aeruginosa* at concentrations of 1.5 × 10^4^ CFU/mL and 1.5 × 10^2^ CFU/mL.

**Figure 13:**
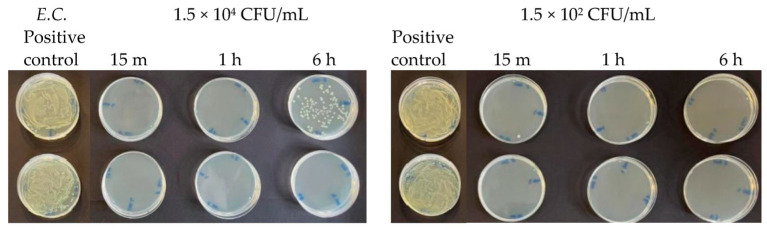
Antibacterial effect of Argirium SUNc^®^ against *Escherichia coli* at concentrations of 1.5 × 10^4^ CFU/mL and 1.5 × 10^2^ CFU/mL.

**Figure 14:**
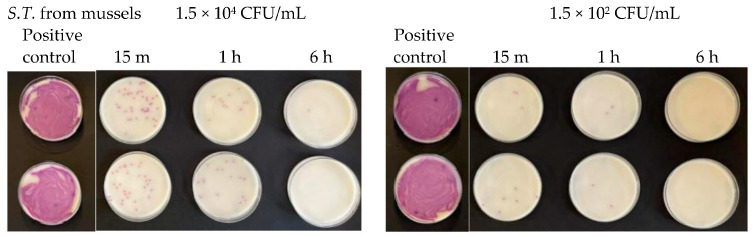
Antibacterial effect of Argirium SUNc^®^ against *Salmonella typhimurium* isolated from mussels at concentrations of 1.5 × 10^4^ CFU/mL and 1.5 × 10^2^ CFU/mL.

**Figure 15:**
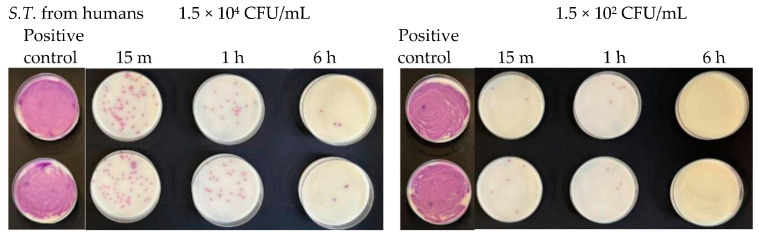
Antibacterial effect of Argirium SUNc^®^ against *Salmonella typhimurium* isolated from humans at concentrations of 1.5 × 10^4^ CFU/mL and 1.5 × 10^2^ CFU/mL.

At microbial concentrations equal to 1.5 × 10^4^ CFU/mL and 1.5 × 10^2^ CFU/mL, a very good inhibitory effect of Argirium SUNc^®^ was reported in all studied Gram-negative bacteria. In fact, for *Pseudomonas aeruginosa*, an almost total inhibition of microbial proliferation was observed in both cases (Figure 12), with inhibitory powers of 99.97% within 15 min after contact and of 100% within 1 h (Table 3). The same condition was observed for *Escherichia coli* (Figure 13), where a limited growth of the bacterium was detected only at a concentration of 1.5 × 10^2^ CFU/mL after 15 min (99.97% inhibition) and at a concentration of 1.5 × 10^4^ CFU/mL after 6 h (99.01% inhibition) (Table 4). For *Salmonella typhimurium* isolated from mussels, an almost total inhibition of microbial growth was observed after 15 min with an inhibitory power of 99.7% (Table 5). After 6 h, at a concentration of 1.5 × 10^2^ CFU/mL, the inhibitory power shown by Argirium SUNc^®^ was 100% and no colonies could be observed on the agar plates (Figure 14). Also, *Salmonella typhimurium* isolated from humans showed its sensitivity to Argirium SUNc^®^ (Figure 15). Inhibitory powers of 99.5% at a concentration of 1.5 × 10^4^ CFU/mL and 99.7% at a concentration of 1.5 × 10^2^ CFU/mL (Table 6) were observed after 15 min. This inhibition had an increasing trend over time, reaching 99.98% at a concentration of 1.5 × 10^4^ CFU/mL and 100% at a concentration of 1.5 × 10^2^ CFU/mL after 6 h.

## 3. Discussion

AgNPs are silver particles that cause the inactivation of the enzymes responsible for the respiration, reproduction, and metabolism of the treated microorganisms. They penetrate in the bacterial cell wall (periplasmic zone), causing bacterial death through the leakage of cytoplasmic contents [31], interaction with its DNA, and interference with normal cellular function [32]. In this study, evaluating their bactericidal activity against the bacteria most commonly found in food and resistant to common antibiotics, it was observed that Argirium SUNc^®^, at a final concentration of 2.2 ppm, partially inactivated all the bacterial strains tested in a suspension with concentration of 1.5 × 10^2^ CFU/mL within 15 min. The results from the studies performed using the same method, but with a higher concentration of the microbial suspensions (1.5 × 10^4^ cells/mL), were quite similar to those at the lower tested concentration. The tested Gram-positive and Gram-negative microorganisms remained viable for significantly longer times in the presence of Argirium SUNc in suspensions with a densities of 1.5 × 10^6^ and 1.5 × 10^7^ CFU/mL. Furthermore, according to other studies [21,33,34], it was observed that Argirium SUNc^®^ had better antimicrobial effects on Gram-negative bacteria than on Gram-positive strains. This lower efficacy observed against Gram-positives is attributed to the difference in the Gram-negative and Gram-positive bacteria surfaces: Gram-negative bacteria have a thin cell membrane (8–12 nm) composed of an inner thin peptidoglycan layer and an outer layer of liposaccharides, with negatively charged lipopolysaccharides, promoting nanoparticle adhesion. Gram-positive bacteria have a thicker membrane (20–80 nm) and negatively charged peptidoglycans that can be an obstacle for Argirium SUNc^®^ penetration, allowing them to be more resistant [35]. The present results are in accordance with those of other studies. According to Kooti et al. (2018), silver caused the disruption of the bacterial cell wall and a loss of cytoplasmic content [36,37,38]. AgNPs were also able to penetrate the bacterial cell, causing death by interacting with its DNA and interfering with normal cellular function, as is confirmed by the research of Mohamed et al. (2020) The available data suggested that AgNPs’ biocidal power is mainly due to the generation of reactive oxygen species, increasing oxidative stress and causing both cytotoxic and genotoxic effects. Dominguez et al. (2020) saw that, at lower and higher concentrations, colloidal silver induced the formation of reactive oxygen species in Gram-negative bacteria and to a much lesser extent in Gram-positive bacteria, which may explain the slower bactericidal activity of AgNPs against Gram-positive microorganisms, also found by us [ Our results are similar to these of Kim et al. (2007) (Argirium vs. NPs 2.2 vs. 7 ppm) and give us reason to support that differences in composition, cell structure, and cell wall thickness between Gram-negative and Gram-positive bacteria may explain why *Escherichia coli*, *Pseudomonas aeruginosa*, and *Salmonella typhimurium* were significantly inhibited by Argirium SUNc^®^, while *Staphylococcus aureus* and *Listeria monocytogenes* were more resistant [23]. Given the results of the present studies, we can say that, due to their bactericidal properties, Argirium SUNc^®^ are practical antimicrobial agents that will be used for years to come.

## 4. Materials and Methods

To test the antibacterial effectiveness of Argirium SUNc^®^, an in vitro study was carried out in which, for each microbial strain considered, four different concentrations (1.5 × 10^7^ CFU/mL, 1.5 × 10^6^ CFU/mL, 1.5 × 10^4^ CFU/mL, and 1.5 × 10^2^ CFU/mL) and three different time intervals (15 min, 1 h, and 6 h) were used. The Argirium SUNc^®^, in the size range of 0.5–3 nm at a concentration of 22 ppm, were provided by the University “G. d’Annunzio” of Chieti-Pescara and synthesized with a patented method (EP-18181873 and structural data are deposited at International Centre for Diffraction Data, ICDD https://www.osti.gov/dataexplorer/biblio/dataset/1191540 (accessed 21 November 2023)). Our nanoparticles are stable for several months in an ultrapure water solution. They are effective against sensitive/resistant bacteria at a very low concentration (<1 ppm), a value much lower than that reported for other silver formulations, and are also very effective at deconstructing mature biofilm (0.6 ppm). Their size (<2 nm) is the smallest of all nanoparticles studied so far, and so are named Argirium Silver Ultra Nano Clusters (Argirium SUNc^®^). *Staphylococcus aureus* ATCC 25923, *Pseudomonas aeruginosa* ATCC 27853, and *Escherichia coli* ATCC 25922 were obtained from American Type Culture Collection, while the two field strains of *Salmonella typhimurium* (one isolated from humans and one from mussels) and one of *Listeria monocytogenes* (isolated from salmon) were provided by the Department of Food Safety Coordination, Istituto Zooprofilattico Sperimentale del Mezzogiorno. All the strains were stored in the freezer at −80 °C. 

### 4.1. Inoculum Preparation

For the reactivation, the strains were seeded in a solid, non-selective nutrient medium called Nutrient agar provided by Biolife (Milan, Italy) and incubated in a thermostat at the optimum growth temperature (37 °C for *Salmonella typhimurium*, *Listeria monocytogenes*, and *Staphylococcus aureus*; 44 °C for *Escherichia coli*; and 25 °C for *Pseudomonas aeruginosa)* and time (24 h of incubation for *Salmonella typhimurium*, *Listeria monocytogenes*, *Staphylococcus aureus*, and *Escherichia coli* and after 48 h for *Pseudomonas aeruginosa)* to evaluate their purity and vitality. Subsequently, for each tested microbial strain, the inoculum was prepared in test tubes with an optical density of 0.5 McFarland standard—1.5 × 10^8^ CFU/mL. 

### 4.2. Laboratory Tests

Starting from the standard inoculum (1.5 × 10^8^ CFU/mL), for each microbial strain, the following concentrations suitable for the purpose of the study were prepared:

1. 1.5 × 10^7^ CFU/mL, made up of 10 µL of Argirium SUNc^®^ at a concentration of 22 ppm, 10 µL of the standard inoculum (1.5 × 10^8^ CFU/mL), and 80 µL of Buffered Peptone Water (BPW);
2. 1.5 × 10^6^ CFU/mL, made up of 10 µL of Argirium SUNc^®^ at a concentration of 22 ppm, 10 µL of previous inoculum (1.5 × 10^7^ CFU/mL), and 80 µL of Buffered Peptone Water (BPW)
3. 1.5 × 10^4^ CFU/mL, made up of 10 µL of Argirium SUNc^®^ at a concentration of 22 ppm, 10 µL of the inoculum at a concentration of 1.5 × 10^5^ CFU/mL, and 80 µL of Buffered Peptone Water (BPW)
4. 1.5 × 10^2^ CFU/mL, made up of 10 µL of Argirium SUNc^®^ at a concentration of 22 ppm, 10 µL of the inoculum at a concentration of 1.5 × 10^3^ CFU/mL, and 80 µL of Buffered Peptone Water (BPW);

All the test tubes containing the prepared solutions were incubated in a thermostat at 37 °C and taken out at different time intervals of 15 min, 1 h, and 6 h to evaluate the reduction in the microbial load and therefore the effectiveness of Argirium SUNc^®^. After respecting the incubation times, from each test tube, 100 µL of solution divided into two aliquots of 50 µL was taken and sowed on two different agar plates containing selective solid culture media (Rapid’*Salmonella* agar OXOID for *Salmonella typhimurium*, Aloa agar Microbiol for *Listeria monocytogenes*, Baird-Parker agar Biolife for *Staphylococcus aureus*, TBX agar Biolife for *Escherichia coli* and *Pseudomonas* CFC agar Biolife for *Pseudomonas aeruginosa*). After sowing, the agar plates were incubated in thermostat at: 37 °C for *Salmonella typhimurium*, *Listeria monocytogenes*, and *Staphylococcus aureus*; 44 °C for *Escherichia coli*; and 25 °C for *Pseudomonas aeruginosa*, and the results were evaluated after 24 h of incubation for *Salmonella typhimurium*, *Listeria monocytogenes*, *Staphylococcus aureus*, and *Escherichia coli* and after 48 h for *Pseudomonas aeruginosa*.

## 5. Conclusions

The spread of microorganisms multi-resistant to antibiotics has reached significant numbers, so much so that the development of substances with innovative antimicrobial activity appears to be an urgent necessity. Argirium SUNc^®^ could be proposed as such, as, to date, there are no formulations on the market that present a broad effectiveness at a low concentrations (<2.5 ppm) and with little environmental impact [39,40,41,42,43,44]. In fact, they are synthesized using patented methods (EU-Pathent) which involve the use of biocompatible ingredients; consequently, the introduction of harmful substances is not foreseen and the concentrations in use do not exceed the limits established by laws. The results obtained from this study provided important information about the Argirium SUNc^®^ spectrum of action against the pathogens and spoilage agents most commonly found in the food chain (*Pseudomonas aeruginosa*, *Salmonella typhimurium*, *Listeria monocytogenes*, *Staphylococcus aureus*, and *Escherichia coli*), harmful both to health and quality aspects. Therefore, they could be used both to formulate bio-gels compatible in the medical field and preparations for environments and equipment sanitization aimed at reducing environmental bacterial contamination. Furthermore, biocompatible functionalized polymers can be produced to be used in food production and conservation, with the aim of limiting bacterial proliferation even in those products whose preparation does not involve heat treatments to reduce the presence of pathogens. This is an aspect that appears particularly promising in order to obtain a valid alternative to traditional antimicrobials in the food industry. However, as can be understood from the data previously shown, so that this perspective can become a real application aimed at reducing the problem of antibiotic resistance, more in-depth studies (not only in vitro but also “in situ”) must be conduct ed on the spectrum of action of these substances and on their real effectiveness (it is important that they are able to act even at relatively low concentrations, so that they can be used in quantities that do not alter food and do not increase production costs for companies), as well as on their ability to inhibit bacterial growth for a prolonged period. In fact, it is essential that the substances used in food preparation and preservation have a broad, but at the same time, highly specific spectrum of action, as their inhibitory action must be aimed at unwanted microorganisms, not at beneficial ones (protechnological microorganisms). 

## 6. Patents

EP-18181873 and structural data are deposited at International Centre for Diffraction Data, ICDD https://doi.org/10.17188/1191540.

## Figures and Tables

**Table 1:** Inhibition percentage (%) of Argirium SUNc^®^ against *Listeria monocytogenes*.

*Listeria monocytogenes*	15 min	1 h	6 h
Microbial concentration	1.5 × 10^2^ CFU/mL		
Plate 1	720 CFU/mL	816 CFU/mL	448 CFU/mL
Plate 2	792 CFU/mL	824 CFU/mL	1004 CFU/mL
Inhibition percentage (%)	49.6	45.3	51.6
Microbial concentration	1.5 × 10^4^ CFU/ml		
Plate 1	99 CFU/mL	98 CFU/mL	31 CFU/mL
Plate 2	108 CFU/mL	89 CFU/mL	28 CFU/mL
Inhibition percentage (%)	99.3	99.4	99.8

**Table 2:** Inhibition percentage (%) of Argirium SUNc^®^ against *Staphylococcus aureus*.

*Staphylococcus aureus*	15 min	1 h	6 h
Microbial concentration	1.5 × 10^2^ CFU/mL		
Plate 1	54 CFU/mL	47 CFU/mL	14 CFU/mL
Plate 2	69 CFU/mL	39 CFU/mL	21 CFU/mL
Inhibition percentage (%)	95.9	97.1	98.8
Microbial concentration	1.5 × 10^4^ CFU/ml		
Plate 1	473 CFU/mL	460 CFU/mL	221 CFU/mL
Plate 2	448 CFU/mL	400 CFU/mL	201 CFU/mL
Inhibition percentage (%)	96.9	97.1	98.59

**Table 3:** Inhibition percentage (%) of Argirium SUNc^®^ against *Pseudomonas aeruginosa*.

*Pseudomonas aeruginosa*	15 min	1 h	6 h
Microbial concentration	1.5 × 10^2^ CFU/mL		
Plate 1	1 CFU/mL	0 CFU/mL	0 CFU/mL
Plate 2	0 CFU/mL	0 CFU/mL	0 CFU/mL
Inhibition percentage (%)	99.97	100	100
Microbial concentration	1.5 × 10^4^ CFU/ml		
Plate 1	10 CFU/mL	0 CFU/mL	0 CFU/mL
Plate 2	14 CFU/mL	0 CFU/mL	0 CFU/mL
Inhibition percentage (%)	99.9	100	100

**Table 4:** Inhibition percentage (%) of Argirium SUNc^®^ against *Escherichia coli*.

*Escherichia coli*	15 min	1 h	6 h
Microbial concentration	1.5 × 10^2^ CFU/mL		
Plate 1	1 CFU/mL	0 CFU/mL	0 CFU/mL
Plate 2	0 CFU/mL	0 CFU/mL	0 CFU/mL
Inhibition percentage (%)	99.97	100	100
Microbial concentration	1.5 × 10^4^ CFU/mL		
Plate 1	0 CFU/mL	0 CFU/mL	0 CFU/mL
Plate 2	0 CFU/mL	0 CFU/mL	149 CFU/mL
Inhibition percentage (%)	100	100	99.01

**Table 5:** Inhibition percentage (%) of Argirium SUNc^®^ against *Salmonella typhimurium* isolated from mussels.

*Salmonella typhimurium* Isolated from Mussels	15 min	1 h	6 h
Microbial concentration	1.5 × 10^2^ CFU/mL		
Plate 1	6 CFU/mL	2 CFU/mL	0 CFU/mL
Plate 2	3 CFU/mL	2 CFU/mL	0 CFU/mL
Inhibition percentage (%)	99.7	99.87	100
Microbial concentration	1.5 × 10^4^ CFU/mL		
Plate 1	44 CFU/mL	20 CFU/mL	0 CFU/mL
Plate 2	32 CFU/mL	13 CFU/mL	1 CFU/mL
Inhibition percentage (%)	99.7	98.89	99.997

**Table 6:** Inhibition percentage (%) of Argirium SUNc^®^ against *Salmonella typhimurium* isolated from humans.

*Salmonella typhimurium* Isolated from Humans	15 min	1 h	6 h
Microbial concentration	1.5 × 10^2^ CFU/mL		
Plate 1	3 CFU/mL	3 CFU/mL	0 CFU/mL
Plate 2	6 CFU/mL	5 CFU/mL	0 CFU/mL
Inhibition percentage (%)	99.7	99.7	100
Microbial concentration	1.5 × 10^4^ CFU/mL		
Plate 1	73 CFU/mL	27 CFU/mL	3 CFU/mL
Plate 2	70 CFU/mL	20 CFU/mL	3 CFU/mL
Inhibition percentage (%)	99.5	99.8	99.98

## Data Availability

Data are contained within the article.
